# Association of Shock Index and Variants with Mortality in Acute Pulmonary Embolism

**DOI:** 10.5811/westjem.48698

**Published:** 2025-12-23

**Authors:** Cameron P. Upchurch, Kristen Sanfilippo, Daphne Lew, Maanasi Samant, Rachel McDonald

**Affiliations:** * University of Vermont Larner College of Medicine, Department of Emergency Medicine, Burlington, Vermont; † University of Vermont Larner College of Medicine, Department of Medicine, Division of Pulmonary and Critical Care Medicine, Burlington, Vermont; ‡ Washington University in St. Louis School of Medicine, Department of Medicine, Division of Hematology, St. Louis, Missouri; § Washington University in St. Louis School of Medicine, Institute for Informatics, Data Science, and Biostatistics, Center for Biostatistics and Data Science, St. Louis, Missouri; || Northwestern University Feinberg School of Medicine, Department of Medicine, Division of Pulmonary and Critical Care Medicine, Chicago, Illinois; # Washington University in St. Louis School of Medicine, Department of Medicine, Division of Pulmonary and Critical Care Medicine, St. Louis, Missouri

## Abstract

**Introduction:**

Pulmonary embolism (PE) is common with potential for morbidity and mortality. Several PE risk-stratification tools exist; however, more granular and patient-specific indicators of potential decompensation or short-term mortality that can be easily obtained are needed for the bedside clinician to further sub-stratify risk and inform management decisions. We sought to determine the association of early emergency department (ED) measurement of the shock index (SI) and SI variants (modified SI, SI to peripheral oxygen saturation ratio, age-adjusted SI, respiratory-adjusted SI, and double product) and mortality among patients with acute PE.

**Methods:**

This was an observational case-control study of adult patients who presented to the ED at a single health system (January 2021–April 2023) and had PE response team (PERT) activation for newly diagnosed acute PE. We evaluated the association of 30-day in-hospital mortality with the SI (heart rate/systolic blood pressure) and variants of the SI—modified SI = heart rate/mean arterial pressure; SI to peripheral oxygen saturation ratio = SI/peripheral oxygen saturation; age-adjusted SI = age x SI; respiratory-adjusted SI = SI x (respiratory rate/10); double product = systolic blood pressure x heart rate—in addition to the Simplified Pulmonary Embolism Severity Index (sPESI) and European Society of Cardiology (ESC) risk schema. We used the area under the receiver operating characteristic curve (AUC) to assess discriminatory efficiency of the SI and each variant with the primary outcome. Multivariable logistic regression measured the association between SI and variants with 30-day mortality.

**Results:**

Of 121 patients included in the study, 12 (9.9%) died. The SI and variants were all significantly different between survivors and non-survivors (*P* < .05), while the sPESI was not different (*P* = .30). The age-adjusted SI had the highest discriminatory efficiency for mortality (AUC 0.82; 95% CI, 0.71–0.93), followed by the SI (AUC 0.78; 0.67–0.89), the SI/peripheral oxygen saturation (AUC 0.77; 0.65–0.90), double product (AUC 0.76; 0.61–0.91), modified SI (AUC 0.75; 0.61–0.90), ESC risk schema (AUC 0.71; 0.52–0.90), and the respiratory-adjusted SI (AUC 0.70; 0.54–0.87).

**Conclusion:**

Among patients presenting to the ED who had a PERT activation for acute PE, the age-adjusted SI had the highest discriminatory efficiency for mortality, followed by the SI and its other variants. Further investigation regarding use of the age-adjusted SI for prognostication of acute PE and implications on PE management is warranted.

## INTRODUCTION

### Background

Venous thromboembolism causes up to 100,000 deaths/year in the United States.^1^ Risk scores such as the Simplified Pulmonary Embolism Severity Index (sPESI) and the European Society of Cardiology (ESC) risk schema are used to identify patients with acute pulmonary embolism (PE) at high risk of death.^2^,^3^ However, simpler risk stratification is needed for the bedside clinician.

The shock index (SI) is a simple tool that can be readily calculated at the bedside (SI = heart rate/systolic blood pressure) and has demonstrated promise in association with patient-centered outcomes. A higher SI is associated with worse outcomes in the generally ill, trauma, and hemorrhaging populations.^4^–^15^ The SI has also been applied to patients with acute PE as an additional indicator of potential for higher mortality.^16^,^17^ There are several variants of the SI: the modified SI (MSI); the SI to peripheral oxygen saturation ratio (SS); the age-adjusted SI (ASI); and respiratory-adjusted shock index (RASI).^18^ The double product (DP) (systolic blood pressure x heart rate) traditionally has been used as an indicator of myocardial strain during stress testing or exercise among patients with chronic cardiovascular disease.^19^ While the DP is not strictly a variant of the SI, it is another easily calculated bedside variable using patient vital signs, although it has not been studied among patients with acute PE. These variants may be better indicators of illness severity and more predictive of patient-centered, short-term outcomes. However, their characterization and application in patients with acute PE is limited.^20^–^26^

### Importance

There is limited research into the SI variants and their association with short-term mortality in patients with acute PE. Risk stratification schema such as the ESC guidelines assign risk categories for patients, but most patients are intermediate risk. Further sub-stratification of individual-level risk may help identify patients who should more likely be admitted to the intensive care unit or receive some form of reperfusion therapy, for example. The use of specific markers of short-term mortality, such as the SI and variants, may further aid bedside clinicians and PE response teams (PERT) in management decisions among patients presenting to the ED with acute PE.

### Goals of this Investigation

We sought to determine the association of early emergency department (ED) measurement of the SI and several SI variants with 30-day mortality for patients with acute PE.

## METHODS

### Study Design and Setting

**Figure 1:**
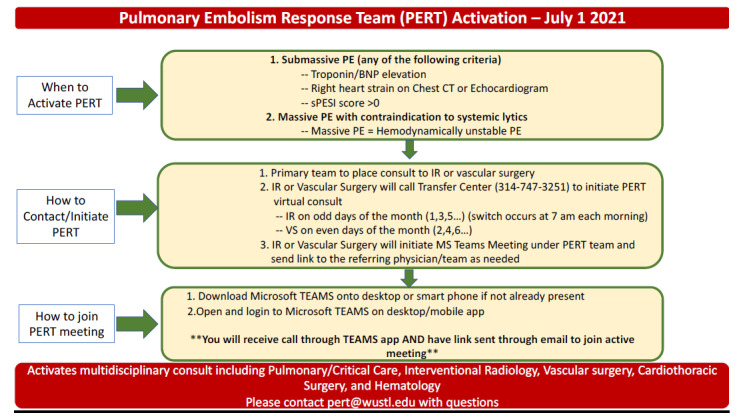
Recommendations and protocol for pulmonary embolism response team activation at Washington University in St. Louis, MO. *BNP*, B-type natriuretic peptide; *CT*, computed tomography; *IR*, interventional radiology; *PERT*, pulmonary embolism response team; *PE*, pulmonary embolism; *sPESI*, Simplified Pulmonary Embolism Severity Index; *VS*, vascular surgery.

This was an observational case-control study of all ED PERT activations between January 2021–April 2023 for patients diagnosed with acute PE at a single health system (Washington University in St. Louis/BJC HealthCare), which encompasses a large, urban, quaternary academic medical center as well as a mix of urban and suburban community medical centers. The PERT activation at our health system was recommended for patients with ESC-defined intermediate- and/or high-risk PE (Figure 1).^3^ We conducted this study in accordance with the Strengthening the Reporting of Observational Studies in Epidemiology guidelines (Supplementary Table 1). Additionally, this study adhered to the following methodologic standards for emergency medicine medical record review studies: abstractors training; case selection criteria; variable definition; performance monitoring; blinding to hypothesis; medical record identification; sampling method; and institutional review board approval.^27^

**Population Health Research Capsule**

What do we already know about this issue?

*Pulmonary embolism (PE) is common with risk for morbidity/mortality. There is need for better predictors of mortality to inform management decisions*.

What was the research question?

*We sought to determine the association of the shock index and several variants with mortality among adults with PE*.

What was the major finding of the study?

*The age-adjusted shock index had the highest discriminatory efficiency for mortality (AUC 0.82; 0.71–0.93)*.

How does this improve population health?

*The age-adjusted shock index may contribute to risk assessment to inform treatment decisions and could be incorporated into future risk prediction rules*.

### Selection of Participants

We included adults (≥ 18 years) if they were diagnosed with acute PE by computed tomography (CT) and had activation of the PERT by the treating emergency clinician. Eligible patients were identified using the health system’s internal PERT database. We excluded patients who underwent PERT activation from the floor or intensive care unit (ICU). Patients were followed from ED presentation until discharge from the hospital or death during index hospitalization, whichever came first. The study was approved by the institutional review board.

### Measurements

Data was abstracted using high-quality chart review standards from the electronic health record by a trained abstractor (clinical research coordinator who completed institutional chart abstraction training) blinded to the study question and hypothesis, and managed using Research Electronic Data Capture hosted at Washington University in St. Louis.^28^,^29^ A second trained abstractor verified data and outcomes for a subset of patients (pre-planned 50% of patients; resulting in 45% of included patients). Initial triage vital signs, baseline patient demographics, labs, and CT findings were used to retroactively calculate the SI, SI variants, sPESI, and ESC risk schema for each patient in the ED. Descriptions of the SI and variants, sPESI, and ESC risk schema are listed in Table 1. All variables extracted from the medical record, and definitions, are provided in Supplementary Table 2.

### Outcome

The primary outcome was 30-day all-cause in-hospital mortality, defined as death in the hospital within 30 days from the time of initial ED triage for the index hospitalization. Patients were followed until either death or discharge. Those discharged alive prior to day 30 were considered to not have experienced the primary outcome.

### Statistical Analysis

We described patient characteristics using descriptive statistics with median and interquartile range (IQR) for continuous variables and counts and percentages for categorical variables. Unadjusted analyses comparing the SI, variants, sPESI, and ESC schema between survivors and non-survivors were performed using chi-squared test for categorical data and the Mann-Whitney *U* test for non-parametric continuous variables. We performed receiver operating characteristic (ROC) analysis and calculated the area under the curve (AUC) for discriminating the primary outcome as well as 95% confidence intervals (CI), and their statistical significance from the null of AUC = 0.5. We used the Youden index to determine the optimal cutoffs for each exploratory variable and its sensitivity as well as specificity for association with mortality.^29^

We used multivariable logistic regression models to assess the association of the SI and variants with the primary outcome, while adjusting for potential confounders based on clinical plausibility (Supplementary Table 2). Final covariates were chosen using forward selection and included the following: age (except in the model assessing the ASI, to avoid redundancy); body mass index (BMI); and Charlson Comorbidity Index, due to their clinical importance and being the variables that were statistically associated with the primary outcome in univariate analyses. Adjusted odds ratios (aOR) and 95% CI were reported. All included patients had complete data for the primary outcome and covariates. We performed Kruskal-Wallis *H* tests to assess for differences between the mean ranks of the SI, variants, and sPESI stratified by ESC risk category. The alpha level was 0.05. Analyses were performed using SPSS Statistics v29 (IBM Corporation, Armonk, NY) and Stata v12.1 (StataCorp, LLC, College Station, TX).

## RESULTS

### Characteristics of Study Subjects

**Figure 2:**
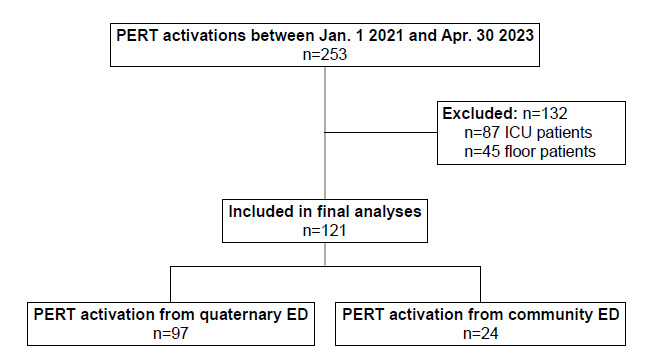
Flow chart with exclusions for retrospective study investigating the association of the shock index and its variants with 30-day in-hospital mortality among adult patients presenting to the emergency department who underwent PERT activation for acute pulmonary embolism. *PERT*, pulmonary embolism response team; *ICU*, intensive care unit; *ED*, emergency department.

During the study period, 121 patients presenting to the ED with acute PE underwent PERT activation and were included in the analysis (Figure 2).

Baseline characteristics of the cohort, stratified by survivors and non-survivors, are summarized in Table 2. Ninety-seven (80.2%) had PERT from the quaternary academic medical center ED, while 24 (19.8%) were from regional community EDs within the health system. A total of 103 patients (85.1%) were classified as “high risk” by sPESI. The PERT recommended anticoagulant therapy alone most frequently (n = 92, 76%), followed by catheter-directed therapy (n = 26, 21.5%), and systemic thrombolysis in one patient (0.8%). Eighty-seven (71.9%) were admitted to an ICU.

### Main Results

The primary outcome of 30-day in-hospital mortality occurred in 12 (9.9%) patients; median length of survival among those who died was 1 day (IQR 0.25–5.5). The SI, MSI, SS, ASI, and RASI were significantly higher, and DP significantly lower, in non-survivors as compared to survivors. However, there was no difference in sPESI classification (“high risk” or sPESI > 0) between survivors and non-survivors (Table 3).

The AUC for mortality was highest for ASI, followed by SI, SS, DP, MSI, ESC, and RASI, which were all significantly different from the null (*P* < .05) (Table 4).

There were significant differences in median SI (*P* < .001), MSI (*P* < .001), SS (*P* < .001), and ASI (*P* < .001) across the ESC risk categories, whereas no significant differences were observed for RASI (*P* = .07), DP (*P* = .52), and sPESI (*P* = .28) (Supplementary Table 3).

## DISCUSSION

This was an observational, case-control study investigating the association between SI and several variants and 30-day all-cause mortality among patients with acute PE who underwent PERT activation. We found that the SI and variants were all significantly different between survivors and non-survivors of acute PE, while sPESI classification was not different between survivors and non-survivors. Furthermore, the ASI had the highest discriminatory efficiency for mortality, followed by the SI, SS, DP, MSI, ESC risk schema, and RASI.

Previous studies have demonstrated similar association of the SI with mortality in acute PE.^16^ However, prior to the present study, there were limited data evaluating the SI variants in acute PE. In a smaller retrospective study, the MSI correlated with markers of right ventricular dysfunction and pulmonary hypertension as well as PESI, and had greater association with mortality as compared to the SI.^30^ Two other retrospective studies had findings similar to our study, demonstrating that ASI had the highest discriminatory efficiency for mortality in patients with acute PE, as compared to the SI and PESI, with AUC similar to that reported in our study at 0.825 and 0.74, respectively.^31^,^32^ Furthermore, to our knowledge, our study is the first to investigate the use of RASI and DP in patients with acute PE.

Risk prediction scores such as the sPESI provide a sensitive tool to identify patients with PE at high risk of death. However, simpler tools that can be readily calculated at bedside promptly after the patient’s arrival, and serially over time, offer the advantage of earlier and repeated risk stratification for this population at risk of early death. While ESC risk schema can inform initial PE risk and treatment decisions, further physiologically based and individualized risk sub-stratification may identify patients among the intermediate- risk group who are at risk for further decompensation despite management based on initial static risk assessment with ESC.

Incorporation of such tools such as the ASI could allow early identification of those who are at risk for worsening and may benefit from more aggressive reperfusion therapies such as systemic thrombolysis or catheter-directed therapies, or the implementation of extracorporeal membrane oxygenation support, if worsening despite previously prescribed indicated therapies. While future study of the application of the ASI among patients with acute PE is needed, our study contributes to the available data that suggest its potential utility given its association with short-term mortality. Future directions may include examining trends in the ASI over time to identify patients who are not adequately responding to systemic anticoagulation alone or certain reperfusion therapies that may warrant management escalation. The ASI could also be considered for inclusion into future risk prediction scores and schema for patients with acute PE.

## LIMITATIONS

Our study has several limitations. First, while we included all patients during our study period who had PERT activation, there are likely patients that had acute PE—potentially less acutely ill given PERT activation at our institution is aimed at ESC-defined intermediate- and high-risk PE patients—who did not have PERT activated and are, therefore, not captured in our study; our findings, therefore, may not be generalizable to patients with ESC-defined low-risk PE. Second, this was a retrospective study with reliance on the vital signs first collected upon arrival to the ED. These vitals may not account for prehospital interventions. Furthermore, given the retrospective design, there is potential for unmeasured confounding. Third, this was a single health system study; thus, the findings require external validation.

Fourth, our primary outcome of short-term mortality occurred in only 12 patients and may have multifaceted etiologies contributing to death beyond the acute PE, and may miss patients discharged alive but who died after discharge. However, similar to sPESI and other PE risk-stratification tools, we feel that indices to aid in potentially predicting all-cause short-term index hospitalization mortality in acute PE are patient-centered. Fifth, therapeutic interventions were at the discretion of the PERT and bedside team, and their potential effect on the primary outcome cannot be determined. Lastly, this study has a limited sample size and patients who experienced the primary outcome, resulting in wide 95% CIs in the adjusted analyses.

## CONCLUSION

In this case-control study of patients presenting to the EDs of a single health system with acute PE, the ASI had the greatest association with in-hospital 30-day mortality, followed by the SI and its other variants. Future studies are needed to assess the clinical and therapeutic role of these findings, with future investigation of the application of the ASI to identify patients that may benefit from closer monitoring in the ICU, reperfusion therapy, and/or extracorporeal membrane oxygenation.

## Supplementary Information







## Figures and Tables

**Table 1:** Description of the shock index and its variants as exploratory variables, as well as the sPESI and ESC risk schema, for retrospective study of their association with 30-day in-hospital mortality for emergency department patients with acute pulmonary embolism.

Exploratory variable	Definition	Computation
Shock Index (SI)	Quotient of the heart rate (HR) and systolic blood pressure (SBP)	SI=HR/SBP
Modified Shock Index (MSI)	Quotient of the HR and mean arterial pressure (MAP)	MSI=HR/MAP
SI to peripheral oxygen saturation ratio (SS)	Quotient of the SI and peripheral oxygen saturation (SpO_2_)	SS=SI/SpO_2_
Age-adjusted SI (ASI)	Product of the SI and age, in years	ASI=SI x age
Respiratory-adjusted SI (RASI)	Product of the SI with the quotient of the respiratory rate (RR), in breaths/min, and 10	RASI=SI x (RR/10)
Double Product (DP)	Product of the SBP and HR	DP=SBP x HR
sPESI	High risk score defined as > 0, or yes to any of the components	Age > 80 years? History of cancer? History of chronic cardiopulmonary disease? HR ≥ 110 bpm? SBP < 100 mm Hg? SpO_2_ < 90%?
ESC risk schema	Low risk, intermediate-low risk, intermediate-high risk, or high risk	Low: sPESI=0 and no RV strain on TTE or CT Intermediate-low: sPESI > 0 and/or RV strain on TTE or CT, but negative troponin Intermediate-high: RV strain on TTE or CT and elevated troponin High: hemodynamic instability

*sPESI*, Simplified Pulmonary Embolism Severity Index; *ESC*, European Society of Cardiology; *PERT*, Pulmonary Embolism Response Team; *SI*, shock index; *MSI*, modified shock index; *RASI*, respiratory-adjusted shock index; *DP*, double product; *HR*, heart rate; *SBP*, systolic blood pressure; *MAP*, mean arterial pressure; *SpO_2_*, peripheral oxygen saturation; *RR*, respiratory rate; *TTE*, transthoracic echocardiogram; *CT*, computed tomography; *bpm*, beats per minute; *mmHg*, millimeters of mercury; *RV*, right ventricular.

**Table 2:** Baseline characteristics for patients who survived versus died from acute pulmonary embolism who had PERT activation, by shock index and its variants and association with 30-day in-hospital mortality.

	All patients (N = 121)	Survivors (n = 109)	Non-survivors (n = 12)
Age, years	62 (54–71)	62 (53–69.5)	68 (61.3–76.5)
Sex, female	58 (47.9)	52 (47.7)	6 (50)
Body Mass Index, kg/m^2^	28.7 (25–35.3)	29.12 (25.5–35.3)	22.8 (17–35.3)
Race			
White	54 (44.6)	50 (45.9)	4 (33.3)
Black	62 (51.2)	54 (49.5)	8 (66.7)
Asian	0 (0)	0 (0)	0
American Indian/Alaska Native	1 (0.8)	1 (0.9)	0
Native Hawaiian/Pacific Islander	1 (0.8)	1 (0.9)	0
Comorbidities			
Charlson Comorbidity Index	4 (2–7)	3 (2–6.5)	7 (5–8.5)
Cancer	40 (33.1)	35 (32.1)	5 (41.7)
Hypertension	78 (64.5)	71 (65.1)	7 (58.3)
Diabetes mellitus	42 (34.7)	38 (34.9)	4 (33.3)
Congestive heart failure	23 (19)	18 (16.5)	5 (41.7)
Vitals			
Heart rate, bpm	113 (99–130)	113 (99–130)	117 (100–131)
Systolic blood pressure, mm Hg	123 (106–143)	127 (111–144)	94 (80–107)
Mean arterial pressure, mm Hg	95 (80.5–106)	96 (85–107)	74 (60–83)
Rrespiratory rate, rpm	20 (18–23)	20 (18–23)	20 (17–27)
SpO_2_	0.92 (0.87–0.97)	0.92 (0.87–0.97)	0.90 (0.86–0.97)
Location of PERT activation			
Quaternary emergency department	97 (80.2)	86 (78.9)	11 (91.7)
Community emergency department	24 (19.8)	23 (21.1)	1 (8.3)
Clot location(s)			
Main pulmonary artery or “saddle”	43 (35.5)	39 (35.8)	4 (33.3)
Segmental	102 (84.3)	92 (84.4)	10 (83.3)
Subsegmental	60 (49.6)	56 (51.4)	4 (33.3)
Clot percent obstruction, %	45.2 (24.8–56.8)	45.8 (25.2–61)	30.7 (3.2–51.8)
Right ventricular strain on CT	88 (72.7)	80 (73.4)	8 (66.7)
Right ventricular strain on TTE (n = 104)	53 (43.8) (n=104)	50 (50.5) (n=99)	3 (60) (n=5)
sPESI score	1 (1–2)	1 (1–2)	2 (0.25–3)
sPESI high risk	103 (85.1)	94 (86.2)	9 (75)
ESC pulmonary embolism severity			
Low risk	3 (2.5)	3 (2.8)	0 (0)
Intermediate-low risk	19 (15.7)	17 (15.6)	2 (16.7)
Intermediate-high risk	72 (59.5)	70 (64.2)	2 (16.7)
High risk	27 (22.3)	19 (17.4)	8 (66.7)
Initial PERT decision			
Anticoagulation alone	92 (76)	82 (75.2)	10 (83.3)
Catheter-directed therapy	26 (21.5)	24 (22)	2 (16.7)
Systemic thrombolysis	1 (0.8)	1 (0.9)	0
No treatment	2 (1.7)	2 (1.8)	0
First anticoagulant used			
Unfractionated heparin	96 (79.3)	86 (78.9)	10 (83.3)
Enoxaparin	22 (18.2)	20 (18.3)	2 (16.7)
Bivalirudin	1 (0.8)	1 (0.9)	0
Apixaban	0	0	0
None	2 (1.7)	2 (1.8)	0
Catheter-directed therapy device used (n = 23)			
Inari	22 (18.2)	21 (19.3)	1 (8.3)
Penumbra	1 (0.8)	0 (0)	1 (8.3)
Intraprocedural systemic thrombolysis given	2 (1.7)	2 (1.8)	0
Supplemental oxygen within 24 hours	95 (78.5)	84 (77.1)	11 (91.7)
NC	74 (61.2)	69 (63.3)	5 (41.7)
NIPPV	8 (6.6)	7 (6.4)	1 (8.3)
HHFNC	6 (5)	5 (4.6)	1 (8.3)
Ventilator	7 (5.8)	3 (2.8)	4 (33.3)
ICU admission	87 (71.9)	80 (73.4)	7 (58.3)
Vasopressors within 24 hours	27 (22.3)	20 (18.3)	7 (58.3)
ECMO used	3 (2.5)	2 (1.8)	1 (8.3)
Hospital length of stay, days	4 (3–8)	5 (3–8)	1 (0.25–5.5)
ICU length of stay, days (n = 87)	2 (1–3) (n = 87)	2 (1–3) (n = 80)	1 (0–4) (n = 7)
Cardiac arrest during admission	12 (9.9)	2 (1.8)	10 (83.3)
30-day in-hospital mortality	12 (9.9)	0 (0)	12 (100)

*PERT*, Pulmonary Embolism Response Team; *bpm*, beats per minute; *mmHg*, millimeters mercury; *rpm*, respirations per minute; *SpO_2_*, peripheral oxygen saturation; *CT*, computed tomography; *TTE*, transthoracic echocardiogram; *sPESI*, Simplified Pulmonary Embolism Severity Index; *ESC*, European Society of Cardiology.

*NC*, nasal cannula; *NIPPV*, non-invasive positive pressure ventilation; *HHFNC*, heated and humidified high flow nasal cannula; *ICU*, intensive care unit; *ECMO*, extracorporeal membrane oxygenation.

**Table 3:** Shock Index and variants, *sPESI, and European Society of Cardiology risk schema of survivors vs non-survivors of patients with acute pulmonary embolism.

	Survivors (n = 109), IQR	Non-survivors (n = 12), IQR	Unadjusted P-value	aOR (95% CI)	Adjusted P-value
SI	0.89 (0.73–1.11)	1.27 (0.98–1.35)	< .001	8.86 (1.42–55.31)	.02
MSI	1.18 (0.98–1.41)	1.60 (1.30–2.12)	< .001	5.96 (1.55–22.92)	.01
SS	0.98 (0.79–1.24)	1.36 (1.10–1.55)	< .001	6.24 (1.35–28.91)	.02
ASI	54 (37.75–69.07)	82.24 (62.25–104.75)	< .001	1.03 (1.01–10.6)	.01
RASI	0.44 (0.35–0.53)	0.56 (0.48–0.69)	.02	18.80 (0.68–521.93)	.08
DP	14208 (11497–17043.50)	10276 (8196–13075)	< .001	1.00 (1.00–1.00)	.04
sPESI high risk	94 (86.2)	9 (75)	.299	n/a	n/a
ESC low risk	3 (2.8)	0 (0)			
ESC intermediate-low risk	17 (15.6)	2 (16.7)			
ESC intermediate-high risk	70 (64.2)	2 (16.7)			
ESC high risk	19 (17.4)	8 (66.7)	< .001	n/a	n/a

* *sPESI*, Simplified Pulmonary Embolism Severity Index; *aOR*, adjusted odds ratio; *ASI*, age-adjusted shock index; *DP*, double product; *ESC*, European Society of Cardiology; *MSI*, modified shock index; *RASI*, respiratory-adjusted shock index; *SI*, shock index; *SS*, shock index to peripheral oxygen saturation.

**Table 4:** Receiver operating characteristic area under the curve for shock index and variants and ESC risk schema in discriminating 30-day in-hospital mortality for adult patients presenting to the emergency department who underwent PERT activation.

	AUC (95% CI)	Optimal cutoff	Sensitivity, %	Specificity, %	AUC compared to null, P-value
SI	0.78 (0.67–0.89)	0.95	91.7	61.5	< .001
MSI	0.75 (0.61–0.90)	1.58	58.3	88.1	< .001
SS	0.77 (0.65–0.90)	1.18	75	71.6	< .001
ASI	0.82 (0.71–0.93)	58.44	91.7	60.6	< .001
RASI	0.70 (0.54–0.87)	0.48	83.3	57.8	.01
DP	0.76 (0.61–0.91)	11615	75	73.4	< .001
ESC Risk Schema	0.71 (0.52–0.90)	3.5	66.7	82.6	.03

*ESC*, European Society of Cardiology; *PERT*, Pulmonary Embolism Response Team; *AUC*, area under the receiver operating characteristic curve; *SI*, shock index; *MSI*, modified shock index; *SS*, shock index to peripheral oxygen saturation; *ASI*, age-adjusted shock index; *RASI*, respiratory-adjusted shock index; *DP*, double product.
