# Supplementary material for: Association of Shock Index and Variants with Mortality in Acute Pulmonary Embolism
Source: West J Emerg Med. 2025 Dec 23;27(1):137–45. doi: 10.5811/westjem.48698 (PMC12815569; doi:10.5811/westjem.48698)
Supplement: Supplementary file 2 [file wjem-27-137-s002.docx]

Supplementary Table 2. Data extraction variables and potential covariates, with their definition and units/options abstracted for this retrospective study evaluation the association of the shock index and its variants with 30-day in-hospital mortality for adult patients presenting the emergency department with acute pulmonary embolism and underwent PERT activation.
