# Supplementary material for: Association of Shock Index and Variants with Mortality in Acute Pulmonary Embolism
Source: West J Emerg Med. 2025 Dec 23;27(1):137–45. doi: 10.5811/westjem.48698 (PMC12815569; doi:10.5811/westjem.48698)
Supplement: Supplementary file 3 [file wjem-27-137-s003.docx]

Supplementary Table 3. Kruskall Wallis H tests to assess for differences between mean ranks of the shock index and its variants between ESC risk categories among adult patients presenting to the emergency department with acute pulmonary embolism and underwent PERT activation. Median values with interquartile ranges are reported for each variant by ESC risk category.


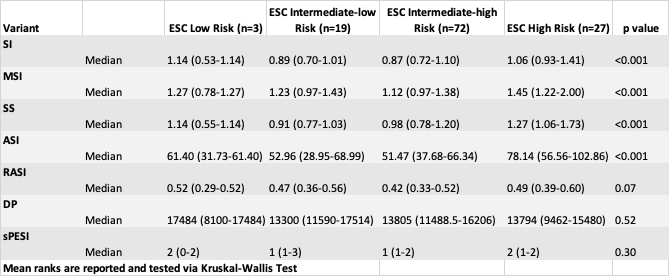


SI, shock index; MSI, modified shock index; SS, shock index to peripheral oxygen saturation; ASI, age-adjusted shock index; RASI, respiratory-adjusted shock index; DP, double product; sPESI, simplified pulmonary embolism severity index; ESC, European Society of Cardiology.
